# Monitoring Parameters During the Immediate Postnatal Transition Period and Inflammatory Markers in the First Two Days After Birth—A Retrospective Data Analysis

**DOI:** 10.3390/children13040529

**Published:** 2026-04-10

**Authors:** Christina H. Wolfsberger, Andreas Hierz, Magdalena Holter, Nariae Baik-Schneditz, Ena Suppan, Bernhard Schwaberger, Gerhard Pichler

**Affiliations:** 1 Division of Neonatology, Department of Pediatrics and Adolescent Medicine, Medical University of Graz, Auenbruggerplatz 34/2, 8036 Graz, Austria; 2 Research Unit for Neonatal Micro- and Macrocirculation, Department of Paediatrics and Adolescent Medicine, Medical University of Graz, 8036 Graz, Austria; 3 Research Unit for Optimization of Postnatal Monitoring for Preterm and Term Neonates, Department of Paediatrics and Adolescent Medicine, Medical University of Graz, 8036 Graz, Austria; 4 Institute for Medical Informatics, Statistics and Documentation, Medical University of Graz, 8036 Graz, Austria

**Keywords:** preterm neonates, term neonates, postnatal transition, near-infrared spectroscopy, regional cerebral oxygen saturation, infectious variables, C-reactive protein

## Abstract

**Highlights:**

**What are the main findings?**

• Early inflammatory markers (CRP and IT ratio) within the first 48 h after birth correlate with cerebral and systemic oxygenation during immediate postnatal transition.
• Microcirculatory alterations may occur despite stable systemic hemodynamics. Inflammatory markers were associated with oxygenation disturbances, while heart rate and mean arterial blood pressure remained unaffected, suggesting subclinical microvascular dysfunction.

**What are the implications of the main findings?**

• Cerebral oxygenation monitoring may reveal subtle vulnerabilities not captured by routine monitoring. Near-infrared spectroscopy (NIRS) detected alterations in cerebral oxygenation that were not reflected by conventional cardiovascular parameters.
• The findings highlight the preventive potential of early risk stratification. Identifying neonates with early inflammatory activation may support targeted monitoring strategies to optimize oxygen delivery during this critical developmental window. These findings suggest that inflammation affects neonatal microcirculation before conventional hemodynamic changes become evident.

**Abstract:**

**Objective:** The fetal-to-neonatal transition is marked by profound cardio-respiratory changes. Infections emerging within the first 48 h after birth may influence early cardiovascular adaptation. We aimed to evaluate the association between early infection/inflammation markers and vital parameters in neonates during the first 15 min after birth. **Methods:** This is a secondary outcome parameter post-hoc analysis of data derived from a prospective observation study. Preterm and term neonates with cerebral oxygen saturation (crSO2) monitoring (INVOS 5100C) during the first 15 min after birth and available inflammatory markers (C-reactive protein [CRP], leukocytes, immature-to-total neutrophils ratio [IT ratio]) within 48 h after birth were included. Heart rate (HR) and arterial oxygen saturation (SpO_2_) were continuously recorded during the first 15 min. Inflammatory markers obtained at 16–24 and 24–48 h after birth were correlated with crSO_2_, SpO_2_, and HR at minute 5, 10 and 15. **Results:** Sixty-eight neonates were included (median (IQR) gestational age 34.0 (32.0; 35.9) weeks, birth weight 1900 (1488; 2542) grams). CRP within the first 24 h correlated negatively with crSO_2_ (r = −0.314; *p* = 0.011) and with SpO_2_ (r = −0.393; *p* = 0.001) at minute 15. IT ratio within 24 h correlated negatively with crSO_2_ at minute 5 (r = −0.367; *p* = 0.005), 10 (r = −0.273; *p* = 0.035), and 15 (r = −0.306; *p* = 0.013), and with SpO_2_ at minute 5 (r = −0.327; *p* = 0.008). IT ratio at 24–48 h correlated negatively with crSO_2_ at minute 15 (r = −0.384, *p* = 0.012). No significant correlations were observed with HR. Leukocytes within the first 24 h after birth correlated negatively with crSO2 at minute 5 (r = −0.265; *p* = 0.046). **Conclusions:** Early inflammatory markers, particularly CRP and the IT ratio, are associated with cerebral and systemic oxygenation during immediate postnatal transition. These findings suggest a potential association between early inflammatory activation and oxygenation dynamics; however, given the observational design and modest correlation strength, the results should be interpreted cautiously and do not allow conclusions regarding causality or underlying mechanisms.

## 1. Introduction

The transition from fetal to neonatal life is a complex and dynamic process, characterized by profound physiological adaptations of the respiratory and cardiovascular systems [1,2]. During this critical period, the neonate must establish effective gas exchange, systemic circulation, and metabolic homeostasis to ensure survival independently of the placenta. Routine monitoring includes pulse oximetry and electrocardiogram (ECG) to assess arterial oxygen saturation (SpO_2_) and heart rate (HR). Near-infrared spectroscopy (NIRS) offers an additional non-invasive tool for continuous monitoring of regional cerebral tissue oxygen saturation (crSO_2_) during the immediate postnatal transition [3,4]. However, whether early inflammatory processes affect oxygenation parameters during this critical period is still unknown.

Early cardiovascular manifestations of neonatal infection can be subtle and challenging to interpret, yet early detection is crucial given the potential for rapid progression to severe morbidity and mortality. Common laboratory markers for suspected infection include C-reactive protein (CRP), leukocyte counts, and immature-to-total neutrophils ratio (IT ratio). However, the interpretation of these parameters within the first 48 h after birth is particularly challenging. An elevated CRP level is a sensitive marker for detecting acute inflammation, which can be of infectious or non-infectious origin. Previous studies using NIRS have demonstrated that inflammatory conditions such as necrotizing enterocolitis are associated with altered regional tissue oxygenation [5]. Furthermore, inflammation-related changes in microcirculation have been described in neonates [6], and elevated CRP levels have been linked to impaired peripheral oxygenation [7], supporting the hypothesis that early inflammatory activation may affect tissue oxygenation. Associations between elevated inflammatory markers and impaired microcirculation have already been demonstrated in neonates: Pichler et al. [7] reported that increased CRP levels after the immediate adaptation phase were linked to reduced peripheral tissue oxygenation, while Binder et al. [8] observed that leukocytosis was associated with decreased perfusion and increased vascular resistance. Wolfsberger et al. [9] found no significant differences in cerebral oxygenation during immediate adaptation phase in preterm infants with fetal inflammatory response syndrome (FIRS), defined as elevated interleukin-6 values (IL-6) above 11 pg/mL compared with those without FIRS.

These findings suggest that even subtle elevations in inflammatory markers during this early period may reflect underlying physiological stress, inflammatory activation or subclinical infection, rather than confirmed infection, potentially impacting the efficiency of oxygen delivery and utilization in vital organs such as the brain.

A more comprehensive understanding of the potential relationship between infection, systemic hemodynamics and cerebral oxygenation may contribute to optimizing neonatal care and improving long-term neurodevelopmental outcomes.

Therefore, the aim of the present study was to investigate correlations between early infectious/inflammatory markers (CRP, leukocyte counts and IT ratio), obtained within the first 24 and 48 h after birth, and SpO_2_, HR and crSO_2_ during the first 15 min after birth.

## 2. Materials and Methods

### 2.1. Design

This study is a post-hoc analysis of secondary outcome parameters derived from a prospective observational study [10] conducted at the Division of Neonatology, Department of Paediatrics and Adolescent Medicine, Medical University of Graz, Austria, between October 2015 and September 2018. The study protocol was approved by the Regional Committee on Biomedical Research Ethics (EC numbers 27-465 ex 14/15), and written informed consent was obtained from parents prior to enrolment of their neonates.

### 2.2. Inclusion and Exclusion Criteria

Eligible participants were preterm and term neonates delivered by Cesarean section who underwent cerebral NIRS monitoring during the first 15 min after birth as a part of the prospective observational study. For the present post-hoc analysis, at least one blood sample for inflammatory markers (CRP, leukocytes, IT ratio) obtained from 16 to 24 h and/or 24 to 48 h after birth was required. The capillary blood sampling was performed as part of routine clinical care at the discretion of the attending neonatologist. Neonates with major congenital malformations were excluded. Only neonates delivered by Cesarean section were included in the original study, as these infants are routinely admitted for standardized postnatal assessment and monitoring in our institution. In contrast, neonates born vaginally are typically not available for continuous structured monitoring immediately after birth.

### 2.3. Monitoring

Antepartum medical history and demographic data (gestational age, birth weight, sex, umbilical artery pH, Apgar scores) were recorded. Delayed cord clamping for 30 s was routinely performed. Neonates were then dried and positioned supine under a radiant warmer. Immediately after birth, a neonatal NIRS sensor (INVOS 5100C Cerebral/Somatic Oximeter Monitor, Medtronic, Minneapolis, MN, USA) was placed on the left fronto-parietal region. Pulse oximetry (IntelliVue MP30 monitor, Koninklijke Philips, The Netherlands) was applied to the neonate’s right hand or wrist to record SpO_2_ and HR. Continuous monitoring was maintained throughout the initial 15 min after birth. Non-invasive blood pressure measurements (systolic arterial blood pressure [SABP], diastolic arterial blood pressure [DABP], and mean arterial blood pressure [MABP]) were obtained from the neonate’s left upper arm at minute 15 after birth using an appropriately sized pneumatic cuff. Rectal body temperature was measured once at minute 15 after birth.

All data were digitally archived using the multichannel system alpha trace digital MM (BESTMedical Systems, Vienna, Austria) for subsequent analysis.

For this analysis, mean SpO_2_, HR and crSO_2_ values were calculated from a 60-s interval at minute 5, 10 and 15 after birth. SpO_2_ and HR values were sampled every second, while crSO_2_ was sampled every eight seconds. As a quality criterion, crSO_2_ values exceeding SpO_2_ were excluded.

### 2.4. Laboratory Analyses

Capillary blood gas analysis (ABL 800 Flex; Radiometer, Brønshøj, Denmark) was performed 12–18 min after birth to determine partial pressure of carbon dioxide (pCO_2_), partial pressure of oxygen (pO_2_), pH and hematocrit values according to the local standard operating procedures.

Capillary blood samples for inflammatory markers (CRP, leukocytes, IT ratio) were collected as part of routine clinical care at the discretion of the attending neonatologist and not according to a predefined study protocol. Available inflammatory parameters routinely assessed at defined time points (16–24 h after birth and 24–48 h after birth) were analyzed in this post-hoc analysis.

### 2.5. Statistical Analysis

Descriptive statistics were calculated to summarize the data, with continuous variables presented as medians and interquartile ranges. Categorical variables were summarized as absolute frequencies and percentages. The relationships between inflammatory markers and vital parameters at 5, 10, and 15 min of life were analyzed using Spearman correlation coefficients, along with their corresponding 95% confidence intervals. The significance level was set to 5% (*p* < 0.05). Statistical analyses were conducted using the R software environment (version 4.5.0). Due to the exploratory nature of this post-hoc analysis and the limited sample size, no multivariable regression analyses were performed, as this would have increased the risk of model overfitting. Therefore, the analysis was restricted to correlation analyses. No adjustment for multiple testing was applied, as the analyses were considered exploratory and hypothesis-generating. Consequently, the reported *p*-values should be interpreted descriptively and with caution.

## 3. Results

**Figure 1:**
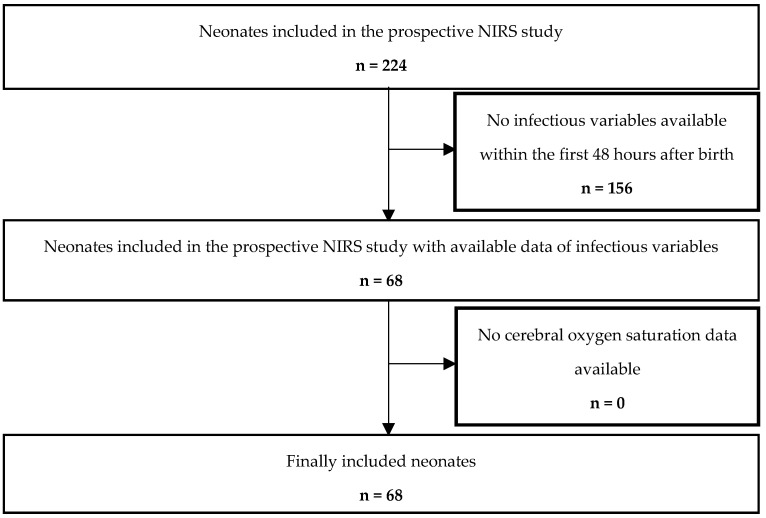
Study flow chart showing the number of included and excluded preterm and term infants.

A total of 224 preterm and term neonates were enrolled in the initial prospective observational study. After applying the inclusion and exclusion criteria, 68 neonates were eligible for this post-hoc analysis (Figure 1, study flow chart). The median (IQR) gestational age of included neonates was 34.0 (32.0; 35.9) weeks, and birth weight was 1900 (1488; 2542) g. Demographic data, monitoring parameters and inflammatory markers are presented in Table 1. The study cohort consisted of 52 preterm and 16 term neonates delivered by Cesarean section. Among preterm neonates, the most common indications for delivery were hypertensive disorders of pregnancy (gestosis, including preeclampsia and HELLP syndrome) in 12 cases (23%), pathological Doppler findings in 10 cases (19%), preterm premature rupture of membranes in 8 cases (15%), preterm labor in 7 cases (13%), and multiple pregnancy in 7 cases (13%). Less frequent indications included amniotic infection syndrome in 4 cases (8%), placental bleeding or abruption in 2 cases (4%), and other indications in 2 cases (4%).

In term neonates, the most frequent indication for Cesarean section was a history of previous Cesarean section in 8 cases (50%), followed by gestosis in 2 cases (13%), placenta previa in 2 cases (13%), intrauterine growth restriction (IUGR) in 2 cases (13%), and pathological cardiotocography (CTG) in 2 cases (13%). One case (6%) was due to multiple pregnancy.

**Figure 2:**
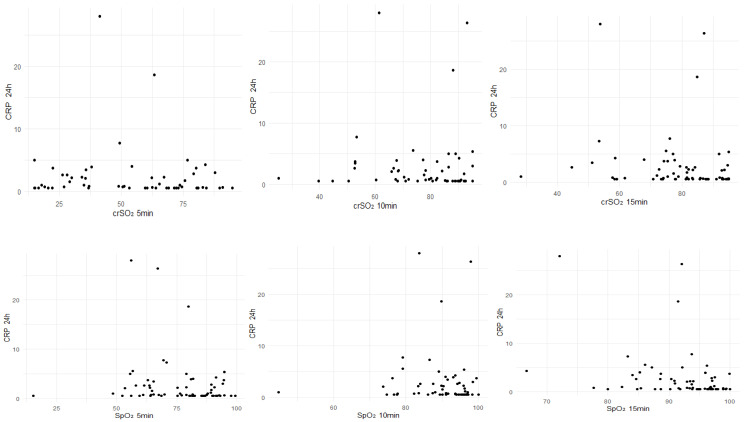
Correlations of CRP with crSO_2_ and SpO_2_.

Correlations between inflammatory markers (CRP, leukocytes and IT ratio) obtained within the first 24 h after birth with SpO_2_, HR, crSO_2_ and MABP during the first 15 min after birth are presented in Table 2 and Figure 2.

CRP within the first 24 h after birth correlated significantly negatively with crSO_2_ (n = 65; r = −0.314; *p* = 0.011) and SpO_2_ (n = 64; r = −0.393; *p* = 0.001) at minute 15 after birth. IT ratio obtained within the first 24 h after birth correlated significantly negatively with crSO_2_ at minutes 5 (n = 57; r = −0.367; *p* = 0.005), 10 (n = 60; r = −0.273; *p* = 0.035) and 15 (n = 65; r = −0.306; *p* = 0.013) and with SpO_2_ at minute 5 (n = 65; r = −0.327; *p* = 0.008). No significant correlations were observed with hemodynamic parameters (HR and MABP).

Correlations between inflammatory markers (CRP, leukocytes and IT ratio), obtained between 24 to 48 h after birth, with SpO_2_, HR, crSO_2_ and MABP during the first 15 min after birth are presented in Table 3. At this time period, only the IT ratio showed a significant negative correlation with crSO_2_ at minute 15 (n = 42; r = −0.384, *p* = 0.012) after birth.

## 4. Discussion

This is the first study to examine potential associations between inflammatory markers in the first days after birth and systemic and cerebral oxygenation, as well as hemodynamic parameters in neonates during the immediate postnatal transition. We found significant correlations between CRP and IT ratio measured 16 to 24 h after birth and both SpO_2_ and crSO_2_ at minute 15 after birth, whereas hemodynamic parameters (HR and MABP) did not reveal any significant associations. Apart from the correlation between IT ratio and crSO_2_ at minute 15 after birth, no significant correlations were observed with inflammatory markers 24 to 48 h after birth and monitoring parameters during the immediate postnatal transition period. However, the present study does not allow for differentiation between infectious and non-infectious inflammatory responses.

Early detection of neonatal infection remains challenging due to the nonspecific nature of initial signs and symptoms [6]. In current practice, diagnosis of early-onset sepsis relies on a combination of clinical evaluation, laboratory markers, and presence of perinatal risk factors. Among the most commonly used laboratory markers are CRP, IT ratio, and leukocyte counts [11], although their diagnostic accuracy depends strongly on the timing of sampling and the aetiology of inflammation.

Several clinical factors may have influenced both inflammatory markers and oxygenation parameters. Gestational age, antenatal steroid exposure, maternal infection, and perinatal interventions such as resuscitation are known to affect neonatal inflammatory responses and cardiovascular adaptation [11,12,13]. As these variables were not controlled for in the present analysis, their potential confounding effects cannot be excluded.

In our cohort, CRP values reached up to 56.0 mg/L within the first 48 h after birth, likely reflecting inflammatory responses triggered by factors such as maternal infection, prolonged rupture of membranes, or the delivery process itself [13,14]. Our findings suggest that this early inflammatory response affects postnatal adaptation primarily at the level of oxygenation (SpO_2_ and crSO_2_), while systemic hemodynamic variables such as HR and MABP remain stable. Pathophysiologically, inflammation-induced reductions in leukocyte deformability and increased endothelial adhesion are known to cause capillary plugging and tissue ischemia—phenomena documented in neonatal sepsis models, where rigid immature neutrophils obstruct capillaries even in the absence of systemic hemodynamic instability [15]. The preservation of systemic hemodynamic stability despite potential microcirculatory impairment may reflect compensatory mechanisms active during the early phase of inflammation. Supporting this interpretation, Pichler et al. demonstrated that neonates with elevated CRP levels exhibited altered peripheral oxygenation despite normal systemic hemodynamic parameters [7], suggesting that microcirculatory disturbances may occur independently of systemic hemodynamic changes.

The association between inflammation and impaired tissue oxygenation is supported by previous studies demonstrating that inflammatory processes can adversely affect neonatal microcirculation. For example, Weidlich et al. [6] showed that early infection in preterm infants is associated with significant alterations in microvascular blood flow, even before overt clinical deterioration becomes apparent. These findings support the concept that inflammatory activation may impair oxygen delivery at the microcirculatory level, which is consistent with the associations observed in our study.

While our finding of a correlation between later inflammatory responses and SpO_2_ aligns with the observations of Wolfsberger et al. of lower SpO_2_ in neonates with FIRS [9], we did not find a similar association with hemodynamic parameters, suggesting a potentially distinct mechanism in our cohort. In detail, they reported no differences in crSO_2_ within the initial 15 min between FIRS and no-FIRS neonates. However, the FIRS group exhibited significantly lower SpO_2_ at minutes 5 and 6, as well as significantly reduced HR at minutes 2 and 4, compared to the non-FIRS group. This discrepancy suggests that FIRS, characterized by elevated perinatal IL-6, mainly affects the first minutes of the immediate transition, whereas prolonged inflammation—captured by CRP and IT ratio within the first days—mainly impacts oxygenation in the later phase of transition. These differences likely reflect the distinct kinetics of inflammatory markers. IL-6 is rapidly induced, whereas CRP rises more slowly [13].

In our cohort, CRP and IT ratio correlated with oxygenation, while leukocytes showed only a transient correlation with crSO_2_ at minute 5 after birth. This pattern suggests that leukocyte counts may indicate a short-lived, non-specific stress response, while CRP and IT ratio reflect the evolving inflammatory burden evident at 15 min. The IT ratio, by contrast, reflects the bone marrow’s early response to infection and is typically considered an earlier indicator than CRP [16], though it is influenced by gestational age and mode of delivery. Leukocyte counts, although widely used, lack specificity [17] and can increase in response to a broad range of stressors, including non-infectious causes such as the stress of birth. Their isolated correlation with crSO_2_ at minute 5 likely reflects this limitation.

Overall, inflammatory markers (CRP, leukocyte, and IT ratio) measured within the first 24 h after birth correlated with SpO_2_ and crSO_2_ during the first 15 min, while those obtained at 24 and 48 h did not. This suggests that early inflammation affects microcirculatory function during the immediate postnatal transition [7], whereas later values reflect systemic responses no longer linked to initial oxygenation dynamics.

### Limitations and Strengths

This study has several limitations that should be considered when interpreting the results. The retrospective nature of the analysis, although based on prospectively collected data, introduces potential biases and limits causal inference. The relatively small number of neonates with available inflammatory markers at 24–48 h after birth reduces statistical power and may explain the lack of significant correlations at the later time point. Although the observed correlations were statistically significant, their strength was modest (r ≈ −0.3), indicating only weak associations. In addition, multiple correlation analyses were performed without adjustment for multiple testing, which increases the risk of Type I error and false-positive findings. Therefore, statistically significant results and clinical relevance of these findings should be interpreted with particular caution. These results are hypothesis-generating and suggest a potential association between early inflammation and oxygenation, but they are not sufficient to support clinical decision-making without further confirmatory studies.

In addition, inflammatory markers were obtained based on clinical indication rather than systematically in all neonates, which may have introduced selection bias toward infants with suspected infection or clinical instability. Only a subset of the original cohort (approximately 30%) had available inflammatory marker data, which may introduce further selection bias and limit the representativeness of the study population.

As an observational study, our findings demonstrate associations but cannot establish causality between inflammatory markers and oxygenation parameters. The study relied primarily on correlation analyses and did not include more robust statistical approaches such as multivariable regression models. Therefore, independent associations and causal relationships cannot be established. The absence of microbiologically confirmed infection limits the ability to distinguish between infectious and non-infectious inflammatory responses.

Furthermore, potentially relevant confounding factors such as gestational age, antenatal steroid exposure, maternal infection, resuscitation measures at birth, and confirmed sepsis diagnoses were not included in the statistical analysis. These factors may influence inflammatory markers and oxygenation parameters and should therefore be considered in future studies using adequately powered multivariable models.

The inclusion of only neonates delivered by Cesarean section reflects local clinical practice, where these infants are routinely available for standardized postnatal monitoring. However, this may limit generalizability, as physiological adaptation differs from vaginally delivered infants.

However, the study also has several notable strengths. Detailed physiological monitoring, including measurements of SpO_2_, crSO_2_, HR, and MABP, enabled comprehensive evaluation of the relationship between inflammatory markers and oxygenation during the critical postnatal transition period. Furthermore, the study also included preterm neonates, a clinically relevant population at high risk for both inflammation and impaired oxygenation, contributing to our understanding of the complex interplay between these factors in this vulnerable population. By examining oxygenation parameters during the immediate postnatal transition period (minute 15 after birth), the study provides valuable insights into the early impact of inflammation on oxygenation.

## 5. Conclusions

This study reveals a correlation between inflammatory markers during the first two days after birth and oxygenation parameters (SpO_2_ and crSO_2_) during the immediate postnatal transition in preterm and term neonates. Elevated CRP levels and IT ratio within the first days after birth were associated with altered oxygenation, suggesting that an early inflammatory response may affect oxygen delivery and consumption during this critical period. Notably, hemodynamic parameters such as HR and MABP did not show significant associations, indicating a more specific association between inflammation and oxygenation rather than systemic cardiovascular instability. The less pronounced correlations observed between inflammatory markers measured 24 to 48 h after birth and monitoring parameters during the immediate postnatal transition period suggest that the impact of inflammation on oxygenation may be most pronounced during the initial stages of postnatal adaptation.

In conclusion, our findings demonstrate weak but statistically significant associations between early inflammatory markers (CRP and IT ratio) and cerebral as well as systemic oxygenation parameters during the immediate postnatal transition. Given the observational design and the modest strength of the correlations, these results should be interpreted as exploratory and hypothesis-generating. No conclusions regarding causality or underlying pathophysiological mechanisms can be drawn from the present data.

## Figures and Tables

**Table 1:** Demographic data, monitoring parameters and infectious variables. Data are presented as median (IQR).

	Median	IQR
Gestational age [weeks]	34.0	32.0; 35.9
Birth weight [g]	1900	1488; 2542
Apgar 1 min	8	8; 9
Apgar 5 min	9	9; 10
Apgar 10 min	10	9; 10
Umbilical cord artery pH	7.32	7.29; 7.34
**Monitoring parameter 5 min after birth**		
SpO_2_ [%]	79	64; 89
HR [bpm]	148	140; 161
crSO_2_ [%]	60	33; 75
**Monitoring parameters 10 min after birth**		
SpO_2_ [%]	91	87; 96
HR [bpm]	148	141; 156
crSO_2_ [%]	82	68; 89
Transcutaneous pCO_2_ [mmHg]	49.0	44.0; 53.8
**Monitoring parameters 15 min after birth**		
SpO_2_ [%]	94	90; 97
HR [bpm]	152	144; 165
crSO_2_ [%]	82	74; 92
SABP [mmHg]	60	56; 65
DABP [mmHg]	34	28; 39
MABP [mmHg]	43	39; 47
Transcutaneous pCO_2_ [mmHg]	50.5	41.5; 57.0
**Capillary blood gas analysis**		
Postnatal age [minutes]	17	16; 19
pH	7.28 h	7.22; 7.31
pCO_2_ [mmHg]	55.1	48.8; 62.7
Haematocrit [%]	55.2	51.9; 59.0
**Infectious variables 16–24 hours after birth**		
CRP [mg/L]	0.9	0.6; 2.9
Leukocytes [/µL]	15,160	9789; 20,110
IT ratio	0.02	0.00; 0.05
**Infectious variables 24–48 h after birth**		
CRP [mg/L]	1.2	0.6; 3.8
Leukocytes [/µL]	11,680	10,570; 20,238
IT ratio	0.02	0.00; 0.04

**Abbreviation**: CRP = C-reactive protein, crSO_2_ = cerebral oxygen saturation, DABP = diastolic arterial blood pressure, HR = heart rate, IT ratio = immature-to-total neutrophils ratio, MABP = mean arterial blood pressure, pCO_2_ = partial pressure of carbon dioxide, SABP = systolic arterial blood pressure, SpO_2_ = arterial oxygen saturation.

**Table 2:** Correlation between infectious variables (CRP, leukocytes and IT ratio) obtained within the first 24 h after birth with SpO_2_, HR, crSO_2_ and MABP during the first 15 min after birth.

	CRP			Leukocytes			IT Ratio		
	*n*	*r*	*p-Value*	*n*	*r*	*p-Value*	*n*	*r*	*p-Value*
**SpO_2_ min 5**	65	−0.196	0.118	65	−0.052	0.681	65	−0.327	0.008
**SpO_2_ min 10**	64	−0.208	0.099	63	−0.060	0.640	63	−0.241	0.057
**SpO_2_ min 15**	64	−0.393	0.001	64	0.081	0.522	64	−0.238	0.059
**HR min 5**	47	−0.177	0.233	47	0.154	0.301	47	−0.083	0.579
**HR min 10**	47	−0.048	0.748	47	−0.023	0.876	47	−0.083	0.579
**HR min 15**	48	0.018	0.901	48	−0.072	0.626	48	−0.135	0.361
**crSO_2_ min 5**	57	−0.130	0.337	57	−0.265	0.046	57	−0.367	0.005
**crSO_2_ min 10**	60	−0.199	0.127	60	−0.147	0.262	60	−0.273	0.035
**crSO_2_ min 15**	65	−0.314	0.011	65	−0.018	0.885	65	−0.306	0.013
**MABP min 15**	58	−0.053	0.691	58	0.030	0.822	58	−0.038	0.778

**Abbreviation**: CRP = C-reactive protein, crSO_2_ = cerebral oxygen saturation, HR = heart rate, IT ratio = immature-to-total neutrophils ratio, MABP = mean arterial blood pressure, SpO_2_ = arterial oxygen saturation.

**Table 3:** Correlations between infectious variables (CRP, leukocytes and IT ratio) obtained between 24 and 48 h after birth with SpO_2_, HR, crSO_2_ and MABP during the first 15 min after birth.

	CRP			Leukocytes			IT Ratio		
	*n*	*r*	*p-Value*	*n*	*r*	*p-Value*	*n*	*r*	*p-Value*
**SpO_2_ min 5**	52	0.117	0.408	53	0.030	0.832	42	−0.077	0.626
**SpO_2_ min 10**	51	0.108	0.453	52	−0.056	0.692	41	−0.104	0.517
**SpO_2_ min 15**	51	−0.136	0.340	52	0.069	0.627	41	−0.204	0.200
**HR min 5**	35	−0.133	0.447	36	0.172	0.316	25	−0.181	0.387
**HR min 10**	36	0.034	0.846	36	−0.202	0.231	25	0.032	0.352
**HR min 15**	35	−0.096	0.577	37	0.049	0.777	26	−0.190	0.880
**crSO_2_ min 5**	46	0.199	0.185	47	−0.092	0.537	38	−0.064	0.702
**crSO_2_ min 10**	50	0.096	0.509	51	−0.142	0.320	41	−0.190	0.233
**crSO_2_ min 15**	52	−0.080	0.575	53	−0.060	0.669	42	−0.384	0.012
**MABP min 15**	45	−0.267	0.077	46	−0.052	0.733	36	−0.191	0.264

**Abbreviation**: CRP = C-reactive protein, crSO_2_ = cerebral oxygen saturation, HR = heart rate, IT ratio = immature-to-total neutrophils ratio, MABP = mean arterial blood pressure, SpO_2_ = arterial oxygen saturation.

## Data Availability

The data are not publicly available but are available from the corresponding author [CHW] upon reasonable request.
